# Bone metastases mimicking Complex Regional Pain Syndrome I: a case report

**DOI:** 10.1186/1752-1947-2-345

**Published:** 2008-11-17

**Authors:** Melchior Huggler, Rudolf Kissling, Florian Brunner

**Affiliations:** 1 Rehaklinik Bellikon, 5454 Bellikon, Switzerland; 2 Department of Physical Medicine and Rheumatology, Balgrist University Hospital, Forchstrasse 340, 8008 Zurich, Switzerland

## Abstract

**Introduction:**

Since there are no valid tools available for the diagnosis of Complex Regional Pain Syndrome I, exclusion of other underlying conditions plays an important role in the diagnostic process.

**Case presentation:**

A 77-year-old Caucasian man was referred with painful swelling and dysfunction of the right knee. Based on the history and clinical presentation, the referring physician assumed a case of Complex Regional Pain Syndrome I. However, after careful evaluation of the differential diagnosis, a metastatic urothelial carcinoma was diagnosed.

**Conclusion:**

Even if the clinical picture resembles Complex Regional Pain Syndrome I, the differential diagnosis must be evaluated carefully.

## Introduction

Complex Regional Pain Syndrome 1 (CRPS 1) is a painful disease with clinical features that include sensory-, sudo- and vasomotor disturbances, trophic changes and impaired motor function [1]. The underlying processes of CRPS 1 still remain unclear and due to the wide spectrum of clinical manifestations, the diagnosis is based on descriptive clinical findings and exclusion of other underlying conditions.

In the past, several diagnostic criteria have been developed. The criteria introduced by the International Association for the Study of Pain (IASP) [2] are the most widely used in clinical practice (see Table 1 for a summary of the IASP criteria). However, the IASP criteria have frequently been criticized because of their moderate sensitivity and low specificity [1,3,4]. Moreover, the poor intraobserver reliability of these criteria casts doubt on their clinical usefulness [5]. In a Delphi experiment, an international panel of experts agreed on a reduced list of relevant diagnostic items [6]. To improve the specificity, another international consensus group proposed a revision of the IASP criteria for CRPS 1 (Budapest criteria) [7]. Only recently, Harden *et al. *[8] published an updated, empirically validated and statistically derived revision of the IASP criteria which shows higher specificity. In contrast to the old version, the new proposed criteria (Budapest criteria) combine signs and symptoms and introduce two sets with different decision rules for use in clinics or research.

**Table 1:** CRPS 1 criteria according to the International Association for the Study of Pain [2]

1. Type 1 is a syndrome that develops after an initiating event
2. Spontaneous pain or allodynia/hyperalgesia occurs, is not limited to the territory of a single peripheral nerve, and is disproportionate to the inciting event
3. There is or has been evidence of edema, skin blood flow abnormality, or abnormal sudomotor activity in the region of the pain since the inciting event
4. This diagnosis is excluded by the existence of conditions that would otherwise account for the degree of pain and dysfunction

For the diagnosis of CRPS 1, criteria 2–4 must be fulfilled.

Nevertheless, one point remains the same in both the old and the new version of the CRPS 1 diagnostic criteria. Clinicians have to rule out other underlying conditions that could present with similar manifestations. This case report of a 77-year-old man with bone metastases illustrates the importance of this item in the criteria list.

## Case presentation

A 77-year-old Caucasian man visited his orthopedic surgeon and complained about persistent right knee pain for the last 2 months. The patient did not remember a specific traumatic event in the past. Upon clinical examination, the surgeon suspected a degenerative meniscus lesion. Since the patient had a pacemaker, further evaluation with magnetic resonance imaging was contraindicated. Intra-articular steroid injection did not lead to a substantial improvement in the symptoms. Based on the available data, it cannot be definitely ruled out that CRPS was absent at that time. The clinical presentation however makes this scenario unlikely.

Since the surgeon supposed that the pain was due to a degenerative meniscus tear, he performed an arthroscopic partial medial and lateral meniscectomy. Shortly thereafter, the patient complained of a dramatic increase in pain intensity and on inspection the surgeon described a newly developed soft tissue swelling, skin color change and hyperhidrosis. He referred the patient to our institution for further evaluation and treatment because he suspected a case of CRPS 1.

**Figure 1:**
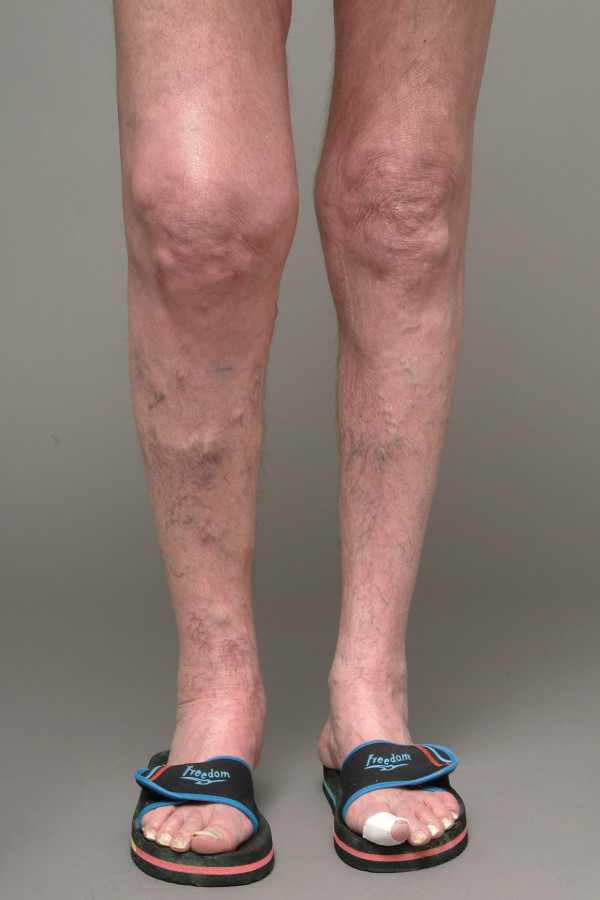
Clinical picture.

Upon examination, the patient was afebrile and complained of consistent pain and soft tissue swelling over the right knee. Due to pain, the patient used two crutches for independent ambulation and was able to walk approximately 30 m. The right knee showed vasomotor (slight rubor, locally increased skin temperature) and sudomotor changes (slight hyperhidrosis) (Figure 1). Active and passive range of motion was painfully limited to flexion/extension of 40°/20°/0°. He demonstrated tenderness on palpation of the medial femoral condyle. Ligamentous stability and meniscal integrity could not be examined due to the pain.

**Figure 2:**
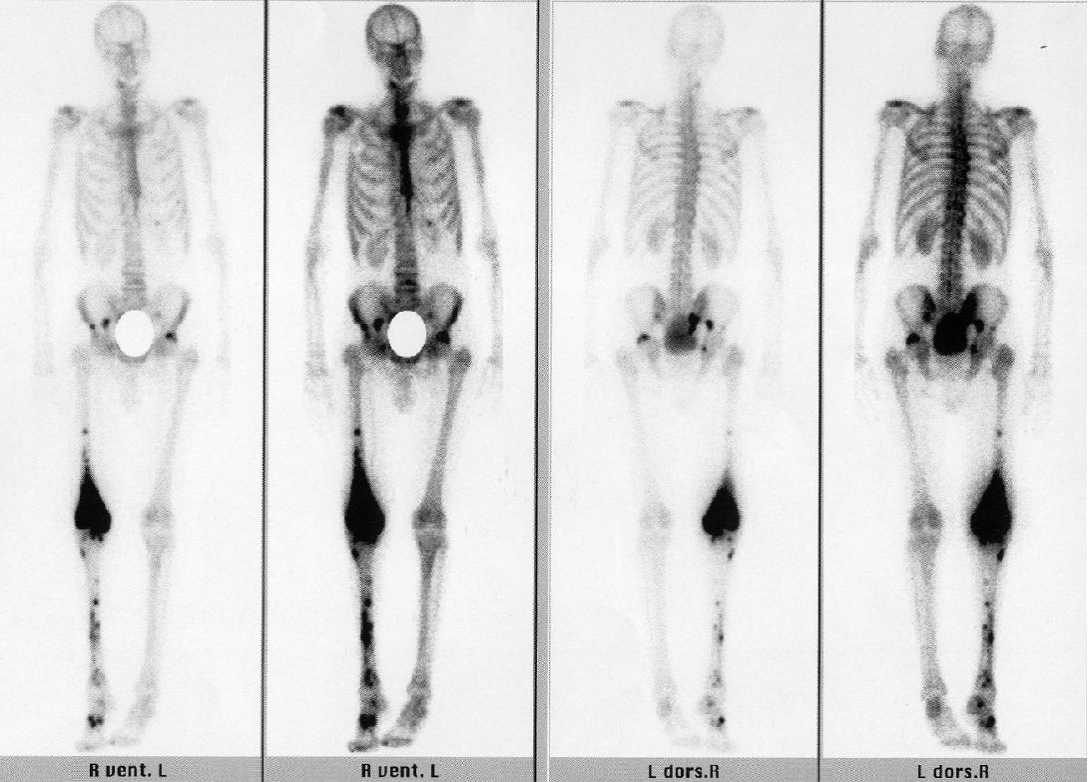
Triple phase bone scan.

Laboratory testing showed the following results: Hb of 12.2 g/dl (<14.0–18.0), ESR 83 mm/hour (8), AP 106 U/liter (40–129), CRP 38.9 mg/liter (<5). Plain radiographs revealed moderate degenerative changes and a moderate intra-articular effusion. Computed tomography (CT) showed some nonspecific trabecular changes in the medial and lateral femoral condyle. Finally, triple phase bone scan with Tc-99m-DPD revealed an increased activity inflow into the distal femoral diaphysis and epiphysis during the perfusion stage. During the second and third phase of the bone scan, multiple enhancements in the distal femur, the right tibia and right hemipelvis were detected (Figure 2). Based on these findings, we concluded that a metastatic process caused the painful swelling and dysfunction. Further evaluation with a biopsy of the femur and cystoscopy revealed the diagnosis of a metastatic urothelial carcinoma. The location of the primary tumor remained unclear and was not further investigated due to the progressive worsening of the patient. After initiating palliative chemotherapy, the patient's condition rapidly deteriorated and he passed away within a few weeks.

## Discussion

This case report emphasizes the importance of carefully evaluating all relevant differential diagnoses as an important step in the diagnostic process of CRPS 1. In this particular case, all points addressing signs and symptoms from the new as well as the old criteria list supported the diagnosis of CRPS 1. See Table 2 for the signs and symptoms used for differential diagnosis of CRPS 1.

**Table 2:** Differential diagnosis of Complex Regional Pain Syndrome 1

Infection
(Para-) Neoplastic
Thrombosis
Gonarthritis: degenerative, septic, crystals (gout, CPPDRA, SLE, reactive)
Avascular bone necrosis
Conversion/self-harm
Dis-/Non-use

CPPD, calcium pyrophosphate disease; RA, rheumatoid arthritis; SLE, systemic lupus erythematosus

As requested in the criteria list, the patient reported an inciting event (arthroscopy), sensory changes (pain) and we found vasomotor and sudomotor changes (edema, change of skin temperature, hyperhidrosis) which are typical for CRPS 1. The clinical examination thus fully supported a diagnosis of CRPS 1.

In order to address the last point of the criteria list and exclude other possible diseases, we continued our differential diagnosis even though the signs and symptoms were very indicative of CRPS 1. Additional testing of blood samples and the CT/bone scan finally revealed that a metastatic malignancy of unknown origin was present. This malignancy accounted for the symptoms and signs found in this patient. Considering the last point of the criteria list, we rejected CRPS 1 as a main diagnosis. Had we stopped our diagnostic process with the points designed to diagnose signs and symptoms, we would have missed the real cause of the patient's complaint.

This case demonstrates the importance of not relying only on inclusion criteria, but of carefully ruling out any other underlying disease. Even if the clinical picture very clearly resembles Complex Regional Pain Syndrome I, the differential diagnosis must be evaluated carefully and all items of the diagnostic criteria for CRPS 1 should be considered.

## Conclusion

The lessons of this case report are twofold. First, this case shows that bone metastases can mimic manifestations compatible with CRPS 1. Second, we believe that this case report is educational showing the possible consequences of premature closure in the diagnostic work-up of CRPS 1. CRPS 1 is usually considered to be a diagnosis by exclusion and the importance of a thorough differential diagnosis addressing all points of the criteria list seems to be crucial.

## Abbreviations

AP: alkaline phosphatase; CPPD: calcium pyrophosphate disease; CRP: C reactive protein; CRPS 1: Complex Regional Pain Syndrome 1; CT: computed tomography; ESR: erythrocyte sedimentation rate; Hb: hemoglobin; IASP: International Association for the Study of Pain; RA: rheumatoid arthritis; SLE: systemic lupus erythematosus; Tc-99m-DPD: technetium-99m-diphosphono-propanodicarboxylic acid.

## Consent

Written informed consent was obtained from the patient's wife for publication of this case report and any accompanying images. A copy of the written consent is available for review by the Editor-in-Chief of this journal.

## Competing interests

The authors declare that they have no competing interests.

## Authors' contributions

MH made the correct diagnosis and wrote the first draft of the manuscript. FB participated in the design of the study, acquired data and wrote the final draft of the manuscript. RK made the correct diagnosis, carried out the clinical management and helped to draft the manuscript. All authors read and approved the final manuscript.
